# Early Placental Angioactive Response to Maternal SARS-CoV-2 Infection: an Immunohistochemical Study

**DOI:** 10.1007/s43032-026-02079-7

**Published:** 2026-03-10

**Authors:** Antonio Carlos de Quadros Junior, Thamirys Cosmo Grillo Fajardo, Maurício Feliciano da Silva, Andrea Cristina Botelho da Silva, Andrea Cristina de Moraes Malinverni, Leonardo Cardili, Estela Bevilacqua, Saulo Duarte Passos

**Affiliations:** 1 Pediatric Infectiology Laboratory, Pediatrics Department, Jundiaí School of Medicine, Jundiaí-SP, Brazil; 2 Laboratory of Molecular and Experimental Pathology I, Department of Pathology, Paulista School of Medicine, Federal University of São Paulo, São Paulo-SP, Brazil; 3 Laboratory of Biology Studies of the Trophoblast and Maternal-Fetal Interaction, University of São Paulo, São Paulo-SP, Brazil

**Keywords:** Placenta, SARS-CoV-2, VEGF, PlGF, NO, COX2

## Abstract

SARS-CoV-2, responsible for COVID-19, affects multiple bodily systems, including placenta. Understanding its effects on placental angioactive factors is vital due to its vascular implications. Herein, we hypothesized that maternal SARS-CoV-2 infection could impact placental morphology and the expression of angioactive and inflammatory factors. The study included 23 pregnant women tested for COVID-19 through reverse transcription polymerase chain reaction (RT-qPCR) and IgG serology around time of delivery and discriminated as: Viremia Group (VG, *n* = 6, maternal positive RT-qPCR, early infection), Serology Group (SG, *n* = 10, maternal positive serology, late infection), and Control Group (CG, *n* = 7, negative for SARS-CoV-2). PCR and immunolocalization of SARS-CoV-2 in the placenta tested negative. Placental morphology was examined using H&E-stained sections, along with immunostaining for vascular endothelial growth factor (VEGF), placental growth factor (PlGF), inducible and endothelial nitric oxide synthase (iNOS, eNOS), and cyclooxygenase 2 (COX2). SG showed a significant higher level of placental morphological changes compared to control samples, which included an increase in syncytial knots and fibrin deposits on the villous surface, and villous peripheral and central infarctions. In contrast, samples from VG exhibited more frequent vascular dilation, congestion, and chorioangiosis. Compared with the control group, immunohistochemical analysis showed significantly higher expression of VEGF (*P* = 0.001), COX2 (*P* = 0.03), and eNOS (*P* = 0.02) in VG. Conversely, in the SG, PlGF levels decreased (*P* = 0.01), while COX2 (*P* = 0.01) and iNOS (*P* = 0.01) levels were elevated. Our data suggests a temporal adaptation of the placenta to maternal SARS-CoV-2 infection, triggering structural, angioactive, and inflammatory responses, enhancing our understanding of potential placental pathways to cope with SARS-CoV-2 infection.

**Supplementary Information:**

The online version contains supplementary material available at 10.1007/s43032-026-02079-7.

## Introduction

Severe acute respiratory syndrome coronavirus 2 (SARS-CoV-2) [1], an enveloped positive-sense single-stranded RNA virus, caused approximately 5 million deaths globally during the 2019–2020 pandemic [2]. Besides its respiratory symptoms, COVID-19 is a systemic disease that affects various organs [3]. Symptom onset typically occurs 5–6 days after exposure, with recovery within two weeks in mild cases. However, in severe cases, this period can extend to several weeks. Notably, post-infection sequelae, including lung fibrosis, myocarditis, and neurological complications, have been observed. Post-infection symptoms, such as fatigue, shortness of breath, and cognitive impairment, can persist for months, eventually for years [4].

The mechanism of infection is mediated by the cell-surface receptor angiotensin-converting enzyme 2 (ACE2, which acts as a gateway for viral entry [5]), and the receptor-binding domain (RBD) of the SARS-CoV-2 spike (S) protein. Neuropilin-1 (NRP1, a vascular endothelial growth factor [VEGF] receptor) has also been identified as an alternative host cell entry receptor for SARS-CoV-2 [6]. This receptor is expressed in many tissues, with particular high levels in endothelial cells and the respiratory and olfactory epithelium [7].

The binding of SARS-CoV-2 to ACE2 disrupts the renin-angiotensin-aldosterone system, which regulates hemodynamic balance and vascular resistance and ultimately determines cardiovascular health and blood pressure. This dysregulation, closely associated with COVID-19 pathogenesis, increases the likelihood of blood clotting, hypertensive episodes, and release of pro-inflammatory factors, contributing significantly to comorbidity-associated mortality, vascular thrombosis, organ-specific morbidity, and multiorgan failure [8, 9].

Imbalances in the production of placental angiogenic factors have been observed in different gestational pathologies in response to various insults such as glycemic, hypertensive, or infectious conditions [10]. Many of these disorders lead to inadequate uterine blood supply and oxidative stress in the placental tissue, with possible fetal outcomes.

Given the potential impact of SARS-CoV-2 infection on inflammation and disruption of the maternal vascular system, it is essential to study the placental response and production of placental angioactive factors in response to this infection.

The specific temporal expression of vascular endothelial growth factor (VEGF) isoforms in the maternal–fetal unit suggests modulatory functions in trophoblast physiology and fetal/maternal vessel development [11]. *VEGF* gene transcription is also induced by stress conditions such as insufficient vascular delivery of oxygenated blood (hypoxia), hormonal inducers such as placental growth factor (PlGF), and inflammatory cytokines. Evidence also shows that VEGF-induced proangiogenic changes, such as vessel dilatation, increased vascular permeability, and angiogenesis, can be at least partly mediated by nitric oxide (NO) [12]. Different nitric oxide synthase isoforms, such as endothelial (eNOS) and inducible (iNOS) can produce this signaling molecule.

PlGF, another critical member of the VEGF family, plays a pivotal role in placental physiology and fetal/maternal vessel development. It is expressed and released by the fetal endothelium and trophoblast cells throughout gestation, and promotes endothelial proliferation, migration, and activation [13]. Furthermore, alterations in PlGF expression levels have been considered a marker of placental disturbances in pathological conditions due to various stimuli, such as hypoxia, nitric oxide, inflammatory cytokines, and growth factors [14].

In the present study, we hypothesized that the early stages of SARS-CoV-2 maternal infection might differentially impact placental morphology and immunohistochemical expression of angioactive (PlGF, VEGF, eNOS) and inflammatory factors (iNOS, cyclooxygenase 2 [COX2]) as a compensatory placental response to maintain local homeostasis.

## Material and Method

### Patients, Sample Collection, and Groups

The cohort consisted of placentas from 23 pregnant women who received prenatal care and delivered at the Jundiaí University Hospital (between 2020 May and June, two months after the World Health Organization (WHO) declared the COVID-19 pandemic [15], and Brazil had already registered approximately 100 confirmed cases). The demographic data and clinical characteristics of the patients are summarized in Table 1. As part of the research procedures, all pregnant women were tested for COVID-19 (real-time polymerase chain reaction [RT-qPCR], and immunoglobulin G [IgG]) pre-delivery, regardless of any symptoms. The examinations were performed between 24 h before and 24 h after birth and included nasopharyngeal swabs and blood samples from the parturient and the newborn. For RT-qPCR, RNA extractions were performed using the QIAamp RNA Mini Kit (Qiagen®, Germantown, USA, #52906), and RT-qPCR tests were used to detect SARS-CoV-2 RNA through Bio-Gene COVID-19® kit (Bioclin Quibasa, Belo Horizonte, Brazil, #K228-1), which utilizes the TaqMan in vitro method. This test detects RNA-dependent RNA polymerase (RdRp) and envelope protein (E) genes. Enzyme-related immunofluorescence assay (ELISA) was performed to detect anti-COVID-19 antibodies of IgG classes using Anti-SARS-Cov-2 IgG ELISA kits (Euroimmun®, São Caetano do Sul, Brazil, #EI 2606-9601 A). All placentas were weighed without cords and membranes, and 2–3 fragments immediately after delivery for PCR and morphological analysis. All laboratory procedures were performed using commercial kits approved by the National Health Surveillance Agency according to the manufacturer’s guidelines.

**Table 1:** Clinical characteristics of pregnant women during the first wave of the COVID-19 pandemic at a public hospital in Brazil

Pregnant’s Characteristics	CG (*n* = 7)	VG (*n* = 6)	SG (*n* = 10)
Maternal age (years old)	29.00 ± 6.32	28.83 ± 6.70	30.3 ± 6.43
Gestational week (weeks ± days)	39.06 ± 1.28 (273.43 ± 8.96 days)	39.21 ± 1.41 (274.50 ± 9.89)	39.03 ± 1.53 (273.20 ± 10.68)
Number of pregnancies *	3 (1–3)	1 and 2 (1–4)	1 and 3 (1–5)
Diabetes mellitus (type II and gestational)	0 (0.00%)	1 (16.67%)	3 (30.00%)
Hypertension	0	2 (33.33%)	1 (10.00%)
Systolic arterial pressure (mmHg)	115.16 ± 6.88	138.33 ± 41.17	119.40 ± 12.31
Diastolic arterial pressure (mmHg)	74.17 ± 10.55	85.50 ± 12.71	76.80 ± 8.24
Obesity	0	2 (33.34%)	1 (10.00%)
Hipotireoidism	0	1 (16.67%)	1 (10.00%)
Apgar index at 5th min	9.26 ± 0.49	9.50 ± 0.55	9.30 ± 0.48
C-section delivery	6 (85.71%)	4 (66.67%)	6 (60.00%)
Birth weight (g)	3035.71 ± 642.91	2935.00 ± 338.73	3038.50 ± 608.13

Data are presented as the mean ± standard deviation. * Number of pregnancies presented as mode (minimal – maximal values). *CG* control group; *VG* viremia group (RT-PCR positive at delivery time); *SG* serology group (IgG positive at delivery time)

Based on the SARS-CoV-2 maternal tests, at the peripartum time, the study groups were determined as follows (Table 1): (i) SARS-CoV-2 positive RT-qPCR group (VG, *n* = 6): pregnant women diagnosed with COVID-19 at delivery time (clinical, laboratory, and virological tests); (ii) SARS-CoV-2-specific IgG positive group (SG, *n* = 10): pregnant women with RT-qPCR test negative and IgG positive tests against SARS-CoV-2 at delivery time, and (iii) Control Group (CG, *n* = 7) consists of pregnant women with negative SARS-CoV-2 tests (clinical, laboratory, and virological tests). The groups were matched for maternal age and gestational age. Patients with other infections or continuous medication use were excluded.

### Morphological Analysis

Immediately after delivery, two placental fragments were collected from different cotyledons, mainly from the central villous areas, and fixed in buffered formalin following routine procedures for inclusion in Histosec (Merck, Darmstadt, Germany). Five-micrometer sections adhered to glass slides treated with a 1% silane solution (3-aminopropyltrimethoxysilane, Sigma Chemical Co., St. Louis, MO, United States) were morphologically characterized by hematoxylin-eosin staining and immunoperoxidase reactions under a light microscope (Axioskop, Zeiss, Germany).

Histopathological examination of the placental tissue included quantification of syncytial knots, perivillous fibrinoid accumulation in spots, and calcification in 10 random fields/placenta samples. Two independent observers performed the measurements at × 20 magnification.

Fibrin deposition (peripheral and central villous infarctions) and vascular villous changes (vascular dilation, vascular congestion, and chorangiosis) were also analyzed based on the frequency of each event in the total of analyzed fields. Areas exhibiting chorangiotic features were identified as those where terminal villi contained ten or more capillaries in non-infarcted regions of the placenta. To avoid bias from comorbidities, evaluations in this study were also conducted excluding patients with DM/DMG and hypertension (three from SG and two from VG).

### Immunoperoxidase

Deparaffinized and dehydrated sections were pretreated with an antigen-unmasking solution in citrate buffer (pH 6.0) for 10 min under pressure-cooking. After cooling down at room temperature, the sections were incubated for 10 min in 3% hydrogen peroxide (H_2_O_2_) and 30 min in 3% BSA (bovine serum albumin) with 0.05% Tween-20 in TBS (Tris-buffered saline; all from Sigma), respectively, for endogenous peroxidase blocking and to prevent non-specific antibody binding. The immunoreactions were performed in batches, with one slide from each placental sample for each antibody tested. The reactions were repeated three times, strictly following the same protocol, reaction times, and reagents.

To block Fc receptors on trophoblast tissues, samples were incubated with 0.05% Human TruStain FcX™ (BioLegend Global Headquarters, San Diego, CA) at room temperature for 5–10 min before staining with the primary antibodies. The sections were then incubated in the following primary antibodies: rabbit polyclonal against inducible nitric oxide (iNOS/NOS2, Sc#7273, Santa Cruz Biotechnology, CA*,* USA)*,* endothelial nitric oxide (eNOS/NOS3, Sc#654, Santa Cruz), and COX2 (*#*SAB5700721, Merck KGaA, Darmstadt, Germany**)**, rabbit monoclonal antibody against PlGF (AB#140639, Abcam, UK), mouse monoclonal antibody against human VEGF (Sc#53,462, Santa Cruz)***,*** and mouse polyclonal antibody against SARS-CoV-2 (#MABX8407, Merck), all diluted 1:100 in TBS-Tween 20, overnight at 4 °C. Goat IgG peroxidase-conjugated against mouse or rabbit (Sigma) was used as a secondary antibody at a 1:100 dilution in TBS-Tween for 1 h at room temperature. Peroxidase was visualized using 3,3'-Diaminobenzidine (DAB) tablets (SIGMAFAST™, Merck) for 5 min. The slides were washed, counterstained with Mayer’s hematoxylin, dried, and mounted with Entellan (Merck). Sections from each placental group were used as negative controls, in which the primary antibody was replaced with Tris-buffered saline or non-immune goat serum.

### Quantitative Analysis of the Immunoperoxidase Reactions

Without knowledge of the groups during counting, four images from each slide of two paraffin blocks were randomly selected for each group and captured, resulting in at least 8 images per group for comparison. The densities of PlGF, VEGF, iNOS, eNOS, and COX2 immunoreactivity were measured and averaged using computer-assisted image analysis (ImageJ, NIH, USA). The microscopic area was calculated as 366,322 µm^2^ and the results were recorded as pixels/µm^2^. Images of the immunoreactions were captured using a × 20 objective in a field of view of 619.52 × 467.20 μm, with an Axioskop 2 Optical Microscope (Carl Zeiss, Germany) equipped with Axio Vision 4.7 software (Carl Zeiss) at a resolution of 1,936 × 1,460 pixels (final resolution of 7 pixels/μm).

### Statistical Analysis

The statistical analyses were conducted using PRISM GraphPad software (GraphPad Software, Inc., San Diego, CA, USA, version 8.0). For morphological characteristics, after confirming the distribution of statistical values, significant differences in syncytial knots, perivillous fibrinoid accumulation in spots, and calcification were analyzed using the Kruskal–Wallis test, followed by Dunn's multiple comparison test. Each characteristic was grouped and averaged, and the results were expressed as mean ± standard deviation. For fibrin deposition (peripheral and central villous infarctions) and vascular villous changes (vascular dilation, vascular congestion, and chorangiosis), the data were presented as the total number of occurrences in the total number of fields analyzed along with their corresponding percentages. Fisher's exact test was used to assess the relationship between the categorical data: CG and VG and CG and SG. For immunoreactions, statistical analysis was performed using the ordinary one-way ANOVA followed by Tukey’s multiple comparisons test. Immunoreactive counts are presented as mean ± standard deviation. For all tested hypotheses, a 2-tailed *p* < 0.05 was considered significant.

## Results

### Patients

The clinical description of the pregnant and non-pregnant groups is in Table 1. Placental fragments from 23 parturient women were analyzed. Six were in an active period of viremia (VG), and ten were infected with SARS-CoV-2 but were no longer actively infected (SG). The remaining seven were not infected with the virus during pregnancy or at birth (CG) (Table 1). All births occurred between weeks 37 and 40, except for one premature birth at 23 weeks (VG). The mean weight of all placentas was 520.87 ± 123.57 g, with no significant difference between the groups. The CG had one vaginal birth, SG had four, and VG had three. All other patients underwent cesarean section. None of the neonates presented with symptoms indicative of SARS-CoV-2 infection.

### Placental Morphological Characteristics

Morphological analysis of the collected fragments revealed that they were primarily composed of chorionic villi. Only 16.5% of the samples included some portion of the decidua. However, the analyses focused solely on the chorionic villi.

**Fig. 1:**
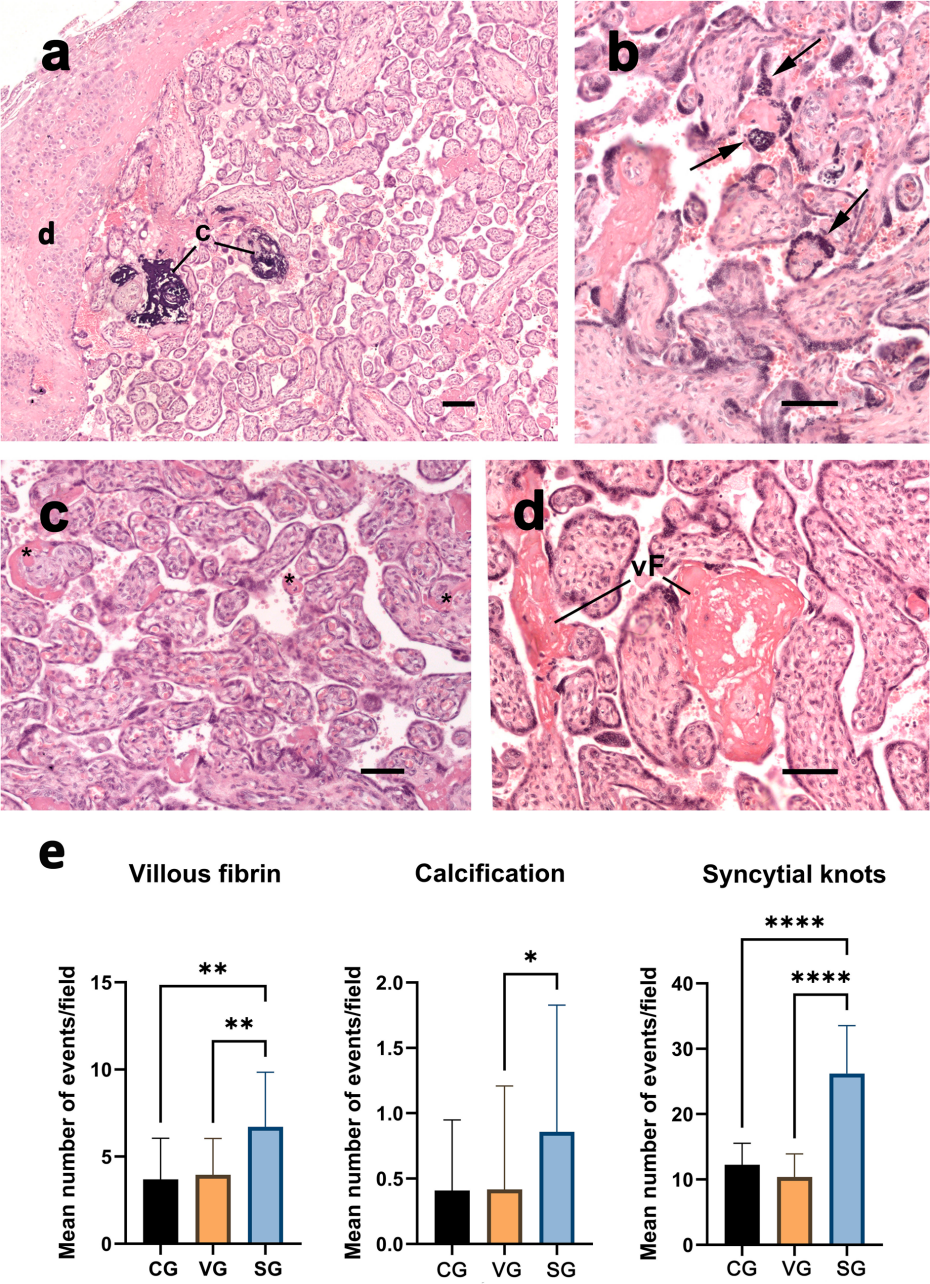
Placental histological parameters. Representative images of (**a**) Calcification (**c**), (**b**) syncytial knots (arrows), and (**c-d**) villous fibrin deposition (*, vF) found in placental samples. H&E staining; Bars = 100 μm. (**e**) These parameters were counted in placental samples from the control (CG), SARS-CoV-2 IgG + (SG), and RT-qPCR + (VG) groups, as described in the Materials and Methods section. Data were averaged, plotted, and represented as mean ± standard deviation. Kruskal–Wallis followed by Dunn’s multiple comparison tests (CG: *n* = 60, VG: *n* = 46 and SG: *n* = 81). *p < 0.05; **p < 0.01; ****p < 0.000

Syncytial knots (*p* < 0.0001) and focal fibrin deposition (*p* = 0.002) at the villous surface were found to be more abundant in the SG compared to the CG. Additionally, significant differences were observed between the VG and SG, with higher values in SG for both syncytial knots (*p* < 0.0001) and fibrin spots (*p* = 0.009). There was no statistically significant difference between CG and VG (Fig. 1e). Calcification levels were only slightly higher in the SG when compared to the VG (*p* = 0.03, Fig. 1e). Except for the number of calcified areas that showed no differences among the groups, all other parameters retained similar differences between infected and control groups after excluding samples with associated comorbidities (Supplementary Table 1).

**Fig. 2:**
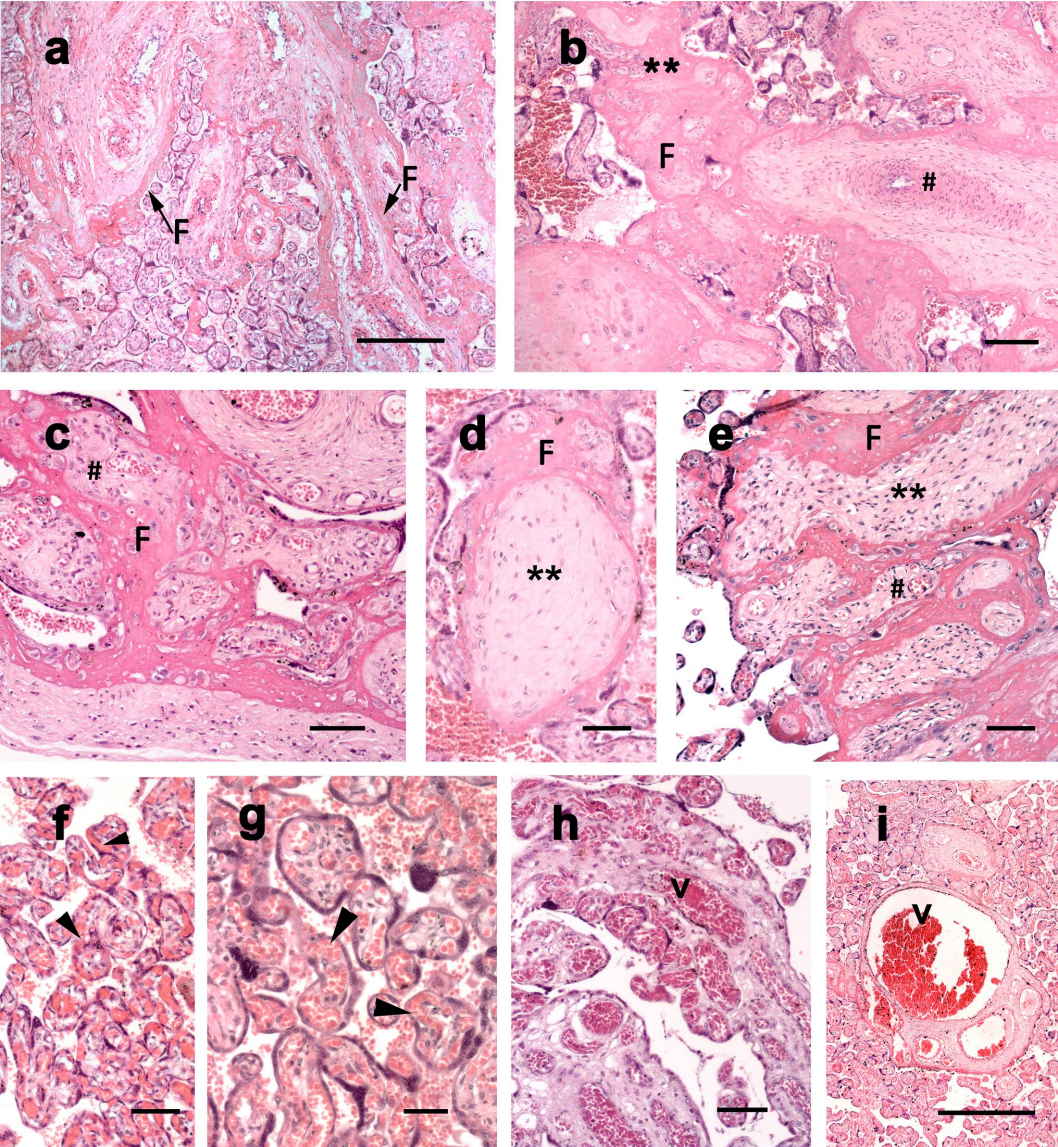
Placental degenerative aspects are more frequently found in infected groups. (**a**-**e**) Peripheral and central villous infarction. (**a**, **c**, **e**) Peripheral fibrin/fibrinoid (F) encases the vascularized (#) parenchyma. (**b**, **d**) In central villous infarction, fibrin/fibrinoid is massively deposited in the parenchyma (**). (**f**-**h**) Vascular villous alterations. (**f**-**g**) Note the abundant capillary network (arrowheads, chorangiosis). (**h**) Vascular congestion (v). (**i**) Vascular dilation (v). Hematoxylin and Eosin staining, bars = 100 μm

Degenerative characteristics of the villi were observed in all groups studied. The relevant changes measured included peripheral and central villous infarction (Fig. 2a-e). Peripheral infarction was characterized by fibrin or fibrinoid encasing the vascularized parenchyma (Fig. 2a, c, e), and central villous infarction by massive deposition of fibrin or fibrinoid in the parenchyma, occluding the local vascularization (Fig. 2b, d). Additionally, vascular alterations in the villi, such as an abundant capillary network (chorangiosis), vascular congestion, and vascular dilation, were also documented (Fig. 2f-i).

Differential incidence of these changes was observed among the groups analyzed (Table 2). Particularly in the SG, degenerative characteristics such as peripheral (*p* = 0.010) and central infarction (*p* = 0.0008) were significantly more frequent than CG. Vascular dilation (*p* = 0.004), vascular congestion (*p* = 0.019) and chorangiosis (*p* = 0.002) were more frequently observed in the VG. Differences were found between the control and infected groups even when samples from patients with comorbidities were excluded (Supplementary Table 1).

**Table 2:** Distribution frequency of histological parameters found in placentas from pregnant women during the first wave of the COVID-19 pandemic in a public hospital in Brazil

Histological parameters	CG: n (%)	VG: *n* (%)	SG: *n* (%)	*p*-value
Peripheral villous infarction	4 (6.7)	7 (15.2)	19 (23.5) #	0.010
Central villous infarction	5 (8.3)	6 (13.0)	26 (32.1) #	0.0008
Vascular dilation	5 (8.3)	14 (30.4) #	9 (11.1)	0.004
Vascular congestion	2 (3.3)	8 (17.4)	0	0.019
Chorangiosis	1 (1.7)	9 (19.6) #	4 (4.9)	0.002

*CG*, Control group; *VG*, Viremia group (RT-qPCR positive at delivery time); *SG*, serology group (IgG positive at delivery time). The parameters were measured as the count of events (n) relative to the total number of microscopic fields analyzed (%). Total number of fields analyzed: CG = 60, VG = 46; SG = 81. Fisher’s exact test. # indicates statistical differences about CG with *P*-value indicated in the last row

### Placental Expression of Angiogenic Factors

All placental samples tested negative for SARS-CoV-2 on RT-qPCR and immune detection (Supplementary Table 2) while the expression of angiogenic factors varied in intensity and cellular origin among the different groups.

**Fig. 3:**
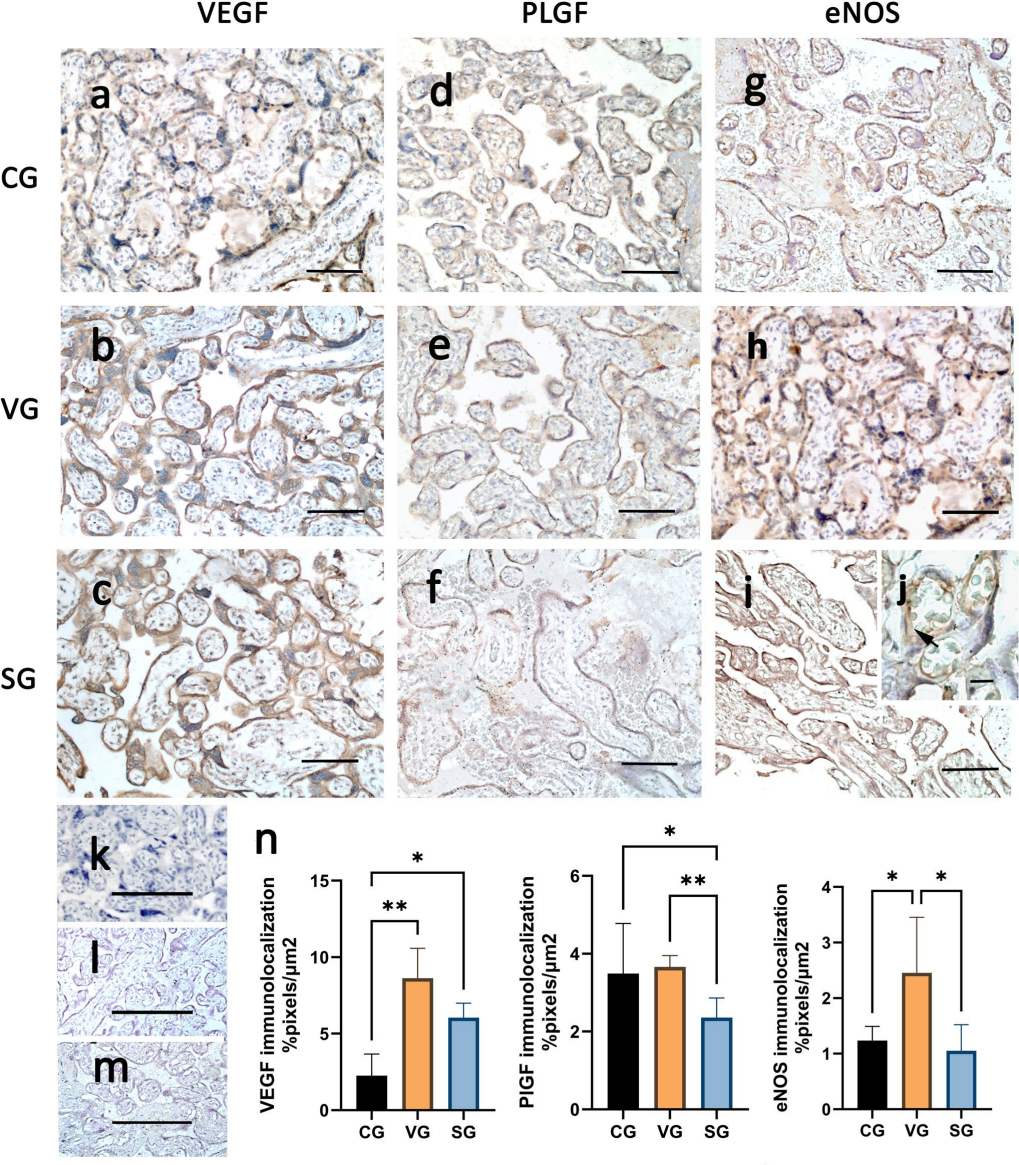
Angioactive responses of VEGF, PlGF and eNOS. Immunolocalization of the angiogenic factors VEGF (a−c), PlGF (d−f) and eNOS (g−j) in the syncytiotrophoblast layer of placentas from control (CG; a, d, g) and infected mothers (VG; b, e, h; SG; c, f, i−j). eNOS immunostaining (arrow) in villous capillaries is shown in (j). Immunoperoxidase and Mayer hematoxylin staining. Bar=100 μm. (k-m). VEGF Immunolocalization graphic (n). Negative control of the immunoreaction, respectively for VEGF, PlGF and eNOS. Quantification of VEGF+, PlGF+ and eNOS+ areas. Data are expressed in mean ± standard deviation of pixels/μm2 (CG: n=60, VG: n=48 and SG: n=81). *p<0.05, **p<0.01. Ordinary one-way ANOVA followed by Tukey’s multiple comparisons test

VEGF was discreetly found in the controls (Fig. 3a) and was strongly labeled in the COVID-19 groups (Fig. 3b-c) in the villous syncytial layer. Quantification showed significant differences between VG (*p* = 0.001) and SG (*p* = 0.03) and controls (CG), as shown in Fig. 3n. As a pro-angiogenic factor, PlGF was also detected in the villous syncytial layer (Fig. 3d-f) in all groups, with decreased expression in the SG compared to the CG (*p* = 0.01) and VG (*p* = 0.007) (Fig. 3n). The immunoreaction for endothelial nitric oxide synthase (eNOS) revealed staining in the villous syncytial layer and endothelial cells (Fig. 3g-j). This reaction was stronger in the VG compared with CG (*p* = 0.020) and SG (*p* = 0.008) (Fig. 3n). No reaction was detected in the immune reaction control samples (Fig. 3k-m).

**Fig. 4:**
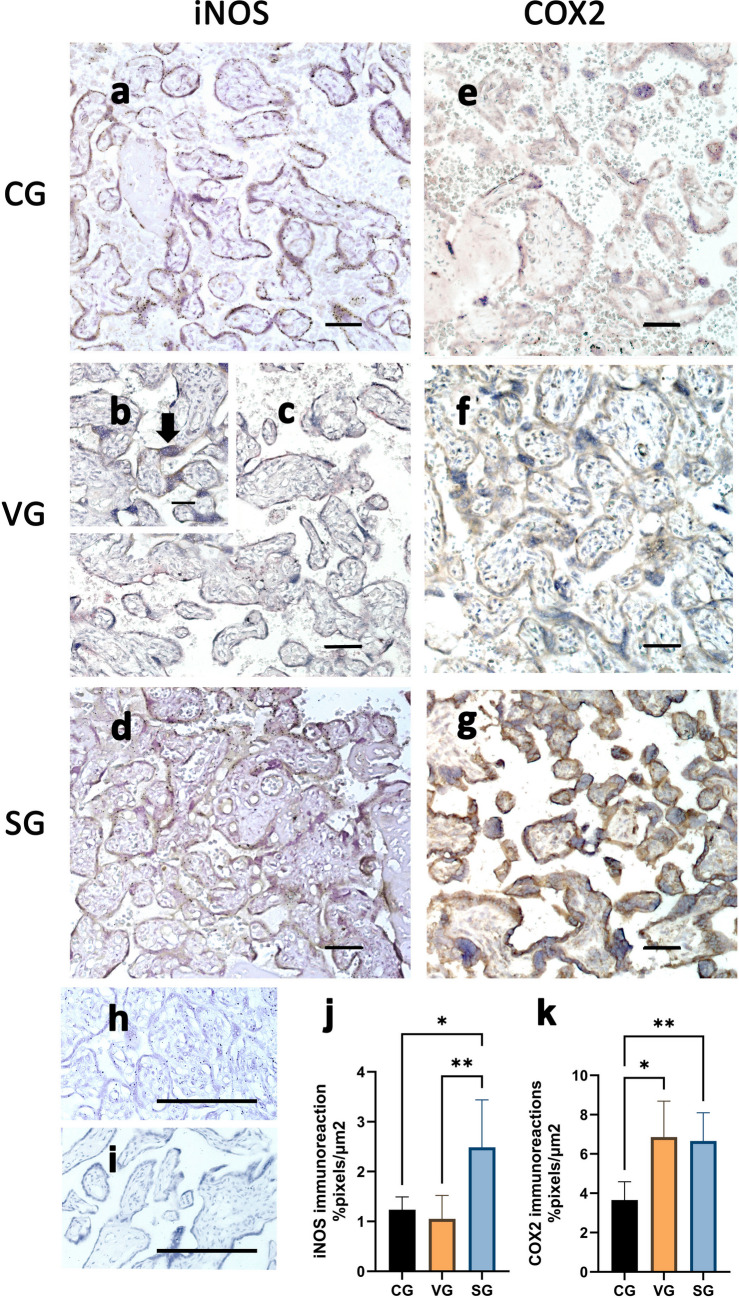
Angioactive responses of iNOS and COX2. Immunolocalization of iNOS (**a**-**d**) and COX2 (**e**-**g**) can be noted in the syncytiotrophoblast layer of placentas from control (CG; **a**, **e**) and infected mothers (VG; **b**-**c**, **f**; SG, **d**, **g**). Panel **b** highlights iNOS immunostaining in a syncytial knot (arrow). (**h**-**i**) Negative control of the immunoreaction, respectively for iNOS and COX2. Immunoperoxidase and Mayer hematoxylin staining. Bar = 100 μm. (**j**-**k**) Quantification of iNOS + and COX2 + areas. Data are expressed in mean ± standard deviation of pixels/μm^2^ (CG: *n* = 60, VG: *n* = 46 and SG: *n* = 81). *p < 0.05, **p < 0.01. Ordinary one-way ANOVA followed by Tukey’s multiple comparisons test

Conversely, the immunolocalization of inducible nitric oxide synthase (iNOS) in the placental samples showed weak staining in the syncytial layer in the control and VG (Fig. 4a-c), and stronger in SG (Fig. 4d). Occasionally, mesenchymal and vascular villous cells were stained, particularly in the SG. Positive brownish COX-2 immunostaining was observed mainly in the syncytiotrophoblast cytoplasm in all groups (Fig. 4e-g), but with greater expression in the infected groups (Fig. 4f-g). No specific immunolocalization was observed in any sections subjected to negative reagent controls in which the primary antibody was omitted (Figs. 4h-i). iNOS quantification (Fig. 4j) revealed significant differences between the CG and SG (*p* = 0.01) and between the SG and VG (*p* = 0.003). COX2 expression was higher in VG (*p* = 0.01) and SG (*p* = 0.006) than in the CG (Fig. 4k). However, no significant differences were observed between the infected groups. We also observed significant differences between the control and infected groups, even after removing samples from patients with comorbidities (see Supplementary Table 3).

## Discussion

In our study, we analyzed the placental morphology and expression of angioactive factors at two different time points during the early stages of SARS-CoV-19 infection: first, during viremia (RT-qPCR-positive pregnant women, early infection) and late infections (RT-qPCR-negative but IgG-positive) at the time of birth. We did not observe any degenerative changes in placental structure in patients who tested positive for viral infection (RT-qPCR-positive). However, vascular dilation and congestion were observed in the capillary villous vasculature. This has been interpreted as a compensatory hemodynamic process or, in some cases, a sign of vascular dysfunction [16]. COVID-19 is linked to blood clotting issues and venous thromboembolism [17, 18], which could potentially affect the maternal–fetal hemodynamic system. Research involving image-based modeling of blood flow has indicated that optimal vascular dilation can enhance oxygen transfer [19]. Therefore, the observed vascular structure may suggest an early adaptive response of the placenta to maternal vascular changes, possibly aimed at increasing the oxygen and nutrient absorption capabilities.

However, later infections (IgG positive patients) completely changed this situation. Infected patients show increased degenerative changes in their placentas, including syncytial knots, intervillous fibrin deposition, central villous infarction, and chorangiosis, suggesting that these changes develop during infection due to chronicity or an organic response. This aligns with other studies that identified increased central and perivillous fibrin deposition, syncytial knotting as placental markers of maternal vascular malperfusion in COVID-19 patients [20–23].

Several studies have identified changes in the placenta due to SARS-CoV-19 infection; the exact cause of these changes remains unclear. Evidence suggests that there may be partial or intermittent obstruction of blood flow to the fetus, such as blockage of the chorionic plate and villous vessels, which can lead to blood clotting and fibrin deposition in the placenta. Perna et al. [24] suggested that the development of these lesions depends on the duration of infection and the pregnancy stage. The authors showed that the villous fibrin deposits and other changes are more pronounced when infection occurs during the third trimester, which supports our results.

Later infections also present with chorangiosis (increased capillaries in the chorionic villi). Although chorangiosis is normal at term, it is exacerbated in pregnancies associated with maternal complications such as chronic prenatal hypoxia [25, 26]. The chorangiosis here is seen as a secondary placental response to SARS-CoV-19 infection, suggesting a need for increased blood and oxygen flow in the chorionic villi, as evidenced by premature increases in vessel dilation and congestion in early infected placentas.

SARS-CoV-2 infection triggers the release of pro-inflammatory cytokines, which play a crucial role in activating immune and blood vessel cells (leukocytes and endothelial cells). This activation is strongly associated with the prothrombotic state during SARS-CoV-2 infection [9]. This condition is also thought to be closely linked to angiogenesis, which promotes the formation of new blood vessels to ensure sufficient oxygen and nutrients during stressful situations [27]. In line with our morphological findings, we examined the protein expression of angioactive factors through immunohistochemical staining for VEGF, PlGF, iNOS, eNOS, and COX2.

In our study, we found high levels of VEGF protein at both stages of infection, indicating continued pro-angiogenic, pro-permeable, and vasodilatory placental activity since the early stages. Several studies suggest that serum levels of angiogenic factors, such as VEGF and PlGF, change in pregnant women with COVID-19, particularly when the infection occurs during the third trimester [28–31]. The placenta actively produces these factors, suggesting active participation in regulating serum levels [13, 14, 32, 34], which aligns with our results and increases VEGF placental expression.

Although PlGF also exhibits angiogenic activity, we observed reduced levels after the acute phase of infection. Previous reports have suggested that in COVID-19, endothelial damage may reduce maternal PlGF levels, similar to preeclampsia. This decrease is linked to pathological interference with the angiotensin receptor ACE2 [34]. Although existing research has focused on maternal serum levels, it is essential to recognize that the placenta is the primary source of PlGF, and any changes in maternal levels may also reflect alterations in placental production [33]. The decrease in placental PlGF protein levels in our study supports this possibility.

The development of blood vessels in the placenta, and their expansion and relaxation, mainly depend on VEGF expression. However, other signaling molecules are involved in or associated with their action. While the exact pathways that control this process are not fully understood, it is well established that VEGF-induced permeability involves the production of nitric oxide (NO) by endothelial nitric oxide synthase (eNOS) [12, 35]. Krause and co-workers demonstrated that nitric oxide, generated by endothelial and inducible nitric oxide synthases (eNOS and iNOS, respectively), plays a vital role in placental physiology and functions as the primary vasodilator [36]. Two primary mechanisms have been suggested for this activity: opening the pores between endothelial cells and forming junctions between them [37].

To address whether nitric oxide could contribute to placental angioactivity in infected mothers, we also analyzed the expression of eNOS and iNOS proteins. Our results showed sustained expression of NOS isoforms, with eNOS predominating in the early stages of maternal infection, followed by an increase in iNOS in the later stages. Although placental NOS isoforms are temporally expressed during gestation [36], an increase in these isoforms, along with an increase in VEGF, may indicate a possible need for increased vasodilation at the maternal–fetal interface in response to the thrombogenic activity of SARS-CoV-2. Interestingly, the later expression of iNOS occurs in parallel with that of COX2, both of which are implicated in the pathophysiology of inflammation and angiogenesis [38, 39]. Similar to eNOS, iNOS is also involved in the production of nitric oxide (NO), but only after induction by an inflammatory condition [40]. COX2 is an essential enzyme in the synthesis of lipid mediators, particularly prostaglandin E2. Enhanced COX2-induced prostaglandin synthesis stimulates angiogenesis, among many other associated processes, including modulation of inflammatory responses, which are also observed following microbial infections [41, 42].

Robust scientific knowledge indicates that immunological dysregulation and inflammation contribute to the development, progression, and severity of COVID-19 [43]. In this context, the presence of factors such as iNOS and COX2, which are not only associated with angiogenesis but are also linked to an inflammatory response, aligns with these findings. It is important to note that the increased expression of these factors occurs in the later stages of infection, after the inflammatory process has already been established.

### Limitations

This study outlined several limiting factors, including sample size, gestational comorbidities during SARS-CoV-2 infection, and the precise timing of the infection. Our samples were collected at a municipal public care hospital in the initial months following the announcement of the COVID-19 pandemic. Our study design aimed to exclude pregnant women diagnosed solely based on symptoms that were not consistently confirmed by molecular tests. Thus, the samples were limited to patients who underwent at least two tests during the peripartum phase (RT-qPCR and IgG), and whose neonates and placentas were also analyzed, which significantly reduced our sampling. Another limiting factor in our study was the inclusion of pregnant women with comorbidities such as diabetes and hypertension. To address this potential issue, we excluded these samples from an additional analysis, as detailed in Supplementary Tables 1 and 3. The results continued to demonstrate statistical differences between the control and infectious groups, similar to those observed in the full sample, suggesting that the changes were associated with the infectious condition. Another critical point in our study was the exact moment of infection, which is usually unclear. Instead of determining a single moment of infection, we defined two periods: early infection for patients who were RT-qPCR-positive and IgG-negative, under the assumption that older infections should also test positive for IgG; and late infection for patients who were IgG-reactive, indicating an infection that had been contracted previously and was only detectable through IgG levels. Our data indicates significant morphological and functional placental adaptations during COVID-19 infection. However, these limitations highlight the need for future studies with larger, more homogeneous samples to validate our findings.

## Conclusion

Our research highlights the critical role of the placenta in the response to maternal SARS-CoV-2 infection. The expression of factors primarily targeting the vascular system, acting on angiogenesis, vasodilation, and inflammation, may be significant in maintaining a balanced maternal-fetal environment, given the potential impact of this virus in causing a prothrombotic condition. These data are crucial for clinical practice and emphasize the necessity for validation in larger representative populations.

## Supplementary Information

Below is the link to the electronic supplementary material.

Supplementary file1 (DOCX 2239 KB)

## Data Availability

All the authors declare consent to availability of data and material.
