# Supplementary material for: Early Placental Angioactive Response to Maternal SARS-CoV-2 Infection: an Immunohistochemical Study
Source: Reprod Sci. 2026 Mar 10;33(3):604–15. doi: 10.1007/s43032-026-02079-7 (PMC13139233; doi:10.1007/s43032-026-02079-7)
Supplement: Supplementary file 1 — Supplementary file1 (DOCX 2239 KB) [file 43032_2026_2079_MOESM1_ESM.docx]

**Table 1.** Clinical characteristics of pregnant women during the first wave of the COVID-19 pandemic at a public hospital in Brazil.

| **Pregnant’s Characteristics** | **CG (n=7)** | **VG (n=6)** | **SG (n=10)** |
| --- | --- | --- | --- |
| Maternal age (years old) | 29.00 ± 6.32 | 28.83 ± 6.70 | 30.3 ± 6.43 |
| Gestational week  (weeks ± days) | 39.06 ± 1.28  (273.43 ± 8.96 days) | 39.21 ± 1.41 (274.50 ± 9.89) | 39.03 ± 1.53 (273.20 ± 10.68) |
| Number of pregnancies * | 3 (1-3) | 1 and 2 (1-4) | 1 and 3 (1-5) |
| Diabetes mellitus (type II and gestational) | 0 (0.00%) | 1 (16.67%) | 3 (30.00%) |
| ~~Obesity~~ | ~~0~~ | ~~2 (33.34%)~~ | ~~1 (10.00%)~~ |
| Hypertension | 0 | 2 (33.33%) | 1 (10.00%) |
| Systolic arterial pressure (mmHg) | 115.16 ± 6.88 | 138.33 ± 41.17 | 119.40 ± 12.31 |
| Diastolic arterial pressure (mmHg) | 74.17 ± 10.55 | 85.50 ± 12.71 | 76.80 ± 8.24 |
| Obesity | 0 | 2 (33.34%) | 1 (10.00%) |
| Hipotireoidism | 0 | 1 (16.67%) | 1 (10.00%) |
| Apgar index at 5^th^ min | 9.26 ± 0.49 | 9.50 ± 0.55 | 9.30 ± 0.48 |
| C-section delivery | 6 (85.71%) | 4 (66.67%) | 6 (60.00%). |
| Birth weight (g) | 3035.71 ± 642.91 | 2935.00 ± 338.73 | 3038.50 ± 608.13 |

Data are presented as the mean ± standard deviation. * Number of pregnancies presented as mode (minimal – maximal values). CG: control group; VG: viremia group (RT-PCR positive at delivery time); SG: serology group (IgG positive at delivery time).

**Table 2.** Distribution frequency of histological parameters found in placentas from pregnant women during the first wave of the COVID-19 pandemic in a public hospital in Brazil.

| **Histological parameters** | **CG: n (%)** | **VG: n (%)** | **SG: n (%)** | **p-value** |
| --- | --- | --- | --- | --- |
| Peripheral villous infarction | 4 (6.7) | 7 (15.2) | 19 (23.5) # | 0.010 |
| Central villous infarction | 5 (8.3) | 6 (13.0) | 26 (32.1) # | 0.0008 |
| Vascular dilation | 5 (8.3) | 14 (30.4) # | 9 (11.1) | 0.004 |
| Vascular congestion | 2 (3.3) | 8 (17.4) | 0 | 0.019 |
| Chorangiosis | 1 (1.7) | 9 (19.6) # | 4 (4.9) | 0.002 |

CG: control group; VG: viremia group (RT-qPCR positive at delivery time); SG: serology group (IgG positive at delivery time). The parameters were measured as the count of events (n) relative to the total number of microscopic fields analyzed (%). Total number of fields analyzed: CG=60, VG=46; SG=81. Fisher’s exact test. # indicates statistical differences about CG with P-value indicated in the last row.
